# Associations of regional-level perceived stress and depression with health-related quality of life in Korean adults: a multilevel analysis of 2017 Korea Community Health Survey data

**DOI:** 10.4178/epih.e2021062

**Published:** 2021-09-08

**Authors:** Eunsu Kim, Min-Ho Shin, Jung-Ho Yang, Soon-Ki Ahn, Baeg-Ju Na, Hae-Sung Nam

**Affiliations:** 1 Department of Public Health, Graduate School, Chungnam National University, Daejeon, Korea; 2 Department of Preventive Medicine, Chonnam National University Medical School, Gwangju, Korea; 3 Public Health and Medical Services Office, Chungnam National University Hospital, Daejeon, Korea; 4 Graduate School of Urban Health, University of Seoul, Seoul, Korea; 5 Department of Preventive Medicine, Chungnam National University College of Medicine, Daejeon, Korea

**Keywords:** Population health, Psychological distress, Depression, Multilevel analysis, Community

## Abstract

**OBJECTIVES:**

We examined the associations of individual and regional-level perceived stress and depression with health-related quality of life (HRQOL) in Korean adults.

**METHODS:**

We used data from the 2017 Korea Community Health Survey, which included 216,713 adults living within 254 municipal districts. As individual-level independent variables, perceived stress (higher vs. lower) and depression (Patient Health Questionnaire-9 ≥10) were defined. Regional-level age-adjusted rates of perceived stress (%) and depression (%) were created for 254 municipal districts and categorized into quartiles to generate regional levels of stress and depression. HRQOL was defined as the individual-level EuroQol 5-dimensional index×100. A multilevel analysis was performed to identify the relationship between individual or regional-level independent variables and individual HRQOL.

**RESULTS:**

In the null model, the proportions of individual variation in the HRQOL explained by region were 1.7% and 2.7% for men and women, respectively. When adjusted with all individual-level variables, regional stress and depression, as well as individual-level perceived stress and depression, were significantly related to HRQOL for both genders. In the full model including all variables, the decrease in HRQOL from the first to the fourth quartile group of regional stress was greater in women (-1.09; 95% confidence interval [CI], -1.87 to -0.31) than in men (-0.65; 95% CI, -1.04 to -0.26).

**CONCLUSIONS:**

Our results suggest that regional-level perceived stress and depression, as well as individual-level perceived stress and depression, are inversely associated with individual HRQOL.

## INTRODUCTION

Health status can be described in terms of self-rated health (SRH) or health-related quality of life (HRQOL), which are assessed using survey questionnaires [1-3]. HRQOL is defined as “a multidimensional concept that includes domains related to physical, mental, emotional, and social functioning.” It focuses on the impact of health status on quality of life [4]. HRQOL can be measured by several indices, such as the Medical Outcomes Study Short Forms (SF-36, SF-12, and SF-6D), the EuroQol 5-dimensional questionnaire (EQ-5D), and the Nottingham Health Profile [5]. Because the EQ-5D and SF-12 are relatively brief and easy to administer [6], both are applicable to large population surveys.

Within regional populations, health can be influenced by both individual-level and area-level factors [7-9]. Individual-level factors refer to all of the individual characteristics that affect an individual’s health level, such as individual socio-demographic characteristics, health behaviors, or physiological conditions. Examples of area-level factors are the physical (natural or built environments) and social (cultural, economic, political, or historical circumstances) environments [7,9-11].

While health outcomes occur at the individual-level, a large proportion of their determinants is usually present at upstream levels that are contextual [11]. When people are exposed to the contextual factors of local environments, they can share similar health conditions to some extent [9]. This may result in the clustering of individual health conditions within a region [9]. Multilevel regression analysis can provide information on how the variance of health outcomes is distributed between the individual and regional levels, and quantifies the clustering of individual health conditions within a region [9].

Psychological factors such as perceived stress and depression are also important health determinants at both population and individual levels [12-16]. The concept of stress has a long history and various dimensions in the health literature [17,18]. Okihiro et al. [18] proposed 3 subcategories of stress: (1) stressors (negative events and conditions such as divorce or job loss); (2) perceived stress (the subjective experience associated with stressors, reflecting the ability to cope); and (3) stress symptoms (physiological and mental reactions) [18,19].

Cohen et al. [20] proposed a heuristic model of the stress process from life events to disease as follows: environmental demands (stressors)→demands appraised as stressful (perceived stress)→negative emotional responses (e.g., depression, anger, or anxiety)→behavioral (poor health decisions and behaviors) and physiological (activation of the sympathetic-adrenal-medullary and hypothalamic-pituitary-adrenal systems) pathways→disease-related physiological changes (e.g., immune, cardiovascular)→increased risk of disease onset or progression [21]. In this model, a negative emotional response occurs when environmental demands (stressors) are perceived to exceed one’s ability to handle them. Negative emotions, such as depression, can affect health via behavioral and physiological pathways. Although the model is unidirectional, there are potential feedback pathways.

A negative correlation between perceived stress [22-24] or depression [24-27] and HRQOL has been reported at the individual-level. However, the effects of regional-level stress or depression on individuals’ health or HRQOL have rarely been studied. Previous studies have focused on the effects of environmental demands on individual health [28,29] or HRQOL [30-33]. Several environmental demands have been identified that worsen an individual’s health or HRQOL, including neighborhood-level deprivation, lack of social capital including social cohesion, poor access to amenities, lack of safety, and poor aesthetic quality [28-33]. Thus, it is necessary to explore the relationship between community psychological indicators and individual health. With reference to the model of Cohen et al. [20], we assumed that the community indicators of perceived stress or depression would reflect the environmental stressors.

We attempted to examine the associations of individual and regional-level perceived stress and depression with individual HRQOL using a multilevel model. To this end, we used data from the Korea Community Health Survey (KCHS) [34], because it includes the above study variables and consists of a large national representative sample (based on 254 municipal districts), enabling a multilevel analysis.

## MATERIALS AND METHODS

### Data

We used data collected in the 2017 KCHS from 228,381 individuals (102,484 men and 125,897 women) living within 254 municipal districts. We excluded 11,668 individuals due to missing data on psychological variables (perceived stress and depression) (n= 852), HRQOL (n= 30), and covariates (n= 10,786). Thus, the final study sample included 216,713 subjects (99,819 men and 116,894 women). Individual-level raw data without personally identifiable information were obtained from the KCHS website (http://chs.cdc.go.kr/).

### Variables

#### Dependent variable

HRQOL was defined as the individual-level EQ-5D score× 100. The EQ-5D is used to measure 5 health factors: mobility, self-care, usual activities, pain/discomfort, and anxiety/depression at 3 response levels: no problem, some problems, and extreme problems. Weights are applied to each of the 5 factors to obtain a single EQ-5D value between 1 and -1, where a score of 0 indicates death. The KCHS data include EQ-5D values weighted as previously described [35] using representative samples from the Korean population.

#### Independent variables

Among the 3 subcategories of stress [18], we selected perceived stress to reflect the concept of stress at the individual-level for this study. Accordingly, individual-level psychological factors included perceived stress and Patient Health Questionnaire-9 (PHQ-9) variables selected from the KCHS results. The individual psychological indicators were averaged and used as proxy indicators for the environmental demands (stressors) of the local community.

The KCHS assessed perceived stress using the question, “How much stress do you feel in your daily life?” The response options were “very much,” “a lot,” “a little bit,” and “hardly any.” Those who responded with “very much” or “a lot” were classified into the high-stress group. Using this variable, regional-level age-adjusted rates of perceived stress were calculated for 254 municipal districts and categorized into quartiles to create a regional-level stress variable (regional stress).

PHQ-9 is a self-reporting test designed as a simple screening tool for depression and its severity [30]. The test consists of 9 items corresponding to the diagnostic criteria for major depressive disorders provided in the Diagnostic and Statistical Manual of Mental Disorders, Fourth Edition. The questions are designed to evaluate the frequency of specific problems within the past 2 weeks; responses are scored on a 4-point scale, where 0= “not at all,” 1= “several days,” 2= “more than 7 days,” and 3= “nearly every day”; thus, the sum of the 9 items ranges from 0 points to 27 points. Following a previous study [36], we defined a PHQ-9 score ≥ 10 as indicating depression for each subject. Using this variable, we calculated regional-level age-adjusted rates of depression for 254 municipal districts and categorized these rates into quartiles to create a regional-level depression variable (regional depression).

#### Covariates

Individual-level KCHS covariates included age (years), body mass index (BMI), marital status (married/cohabitating, divorced/separated/widowed, or never married), education (less than middle school, middle school, high school, or college or more), monthly household income (< 1.00 million, 1.00-1.99 million, 2.00-2.99 million, 3.00-3.99 million, or ≥ 4.00 million Korean won [KRW]), residential area (rural or urban), current smoking, high-risk drinking, physical activity (moderate physical activity for ≥ 30 minutes 5 times per week or vigorous physical activity ≥ 20 minutes 3 times per week) [10], and diagnosed hypertension, diabetes, or arthritis. BMI was calculated as self-reported weight (kg) divided by height squared (m^2^) and converted into 4 categories according to World Health Organization (WHO) Asian classifications [37] as underweight (< 18.5 kg/m^2^), normal (18.5-22.9 kg/m^2^), overweight (23.0-24.9 kg/m^2^), or obese (≥ 25.0 kg/m^2^). The normal-weight group was used as a reference group.

### Statistical analysis

We compared general characteristics between genders in terms of frequency (%) or mean± standard deviation (SD) using the chi-square test or Student t-test. A multilevel analysis was performed to identify the relationship between individual-level or regionallevel psychological factors and individual HRQOL. Since the KCHS contains complex sample survey data, the multilevel analysis was performed by applying the sample weights. This analysis was conducted after subjects were stratified into men and women due to differences in test results according to gender. The intraclass correlation (ICC) was measured as follows: ICC= (municipal districtlevel variation in HRQOL)/(municipal district-level variation in HRQOL+individual-level variation in HRQOL). All statistical analyses were performed using Stata version 14.0 (StataCorp., College Station, TX, USA), and statistical significance was evaluated at a level of p-value < 0.05.

### Ethics statement

The Institutional Review Board (IRB) of Chungnam National University exempted this study from ethical review (IRB No. 201910-SB-176-01).

## RESULTS

Table 1 presents the general characteristics of the study subjects. The proportion of women was 53.9%, and the average age was 52.9± 17.1 years. The average HRQOL score was 94± 12, and it was higher among men (95± 11) than among women (92± 13). The percentage of subjects reporting higher perceived stress and PHQ-9≥ 10 were 23.8% and 3.2%, respectively, and these proportions were both higher in women than in men.

Men and women showed significantly different distributions of covariates such as BMI, marital status, education, household income, residential area, current smoking, high-risk drinking, physical activity, and diagnosed hypertension, diabetes, or arthritis. Women were more likely to be divorced or widowed, less educated (a higher percentage of those with less than a middle-school education), and to have lower household incomes (a higher percentage of those with an income less than 1.00 million KRW) than men. There were substantial differences in current smoking and high-risk drinking rates between men and women. Men were more likely to be current smokers and high-risk drinkers than women. Men had a higher rate of physical activity than women, while their prevalence of overweight or obesity was higher than that of women. Men had a higher prevalence of diabetes and a lower prevalence of arthritis than women. The gender differences in the urban residence rate and the prevalence of hypertension were only about 1% each (Table 1).

Factors related to EQ-5D for men and women according to the weighted multilevel model results are shown in Table 2. The null model analyzed regional variation in EQ-5D when all independent variables and covariates were excluded; significant variation in EQ-5D was observed among municipal districts (p< 0.001). The ICC value was calculated as 0.017 for men, which indicates that 1.7% of the total EQ-5D variance was explained by municipal districts; women showed a higher ICC value (0.027). Model I included individual-level covariates including perceived stress and depression; the ICC values decreased to 0.007 and 0.012 for men and women, respectively, compared to those of the null model. Model II included regional stress (first to fourth quartiles) and individual-level covariates; the ICC values were 0.006 for men and 0.010 for women. Model III included regional depression (first to fourth quartiles) and individual-level covariates; the ICC values were 0.006 for men and 0.013 for women. Model IV (full model) included regional stress, regional depression and covariates; it produced no significant additional reduction in ICC for either gender, compared to those of model I.

In the full model, all covariates except residential area (rural or urban) were significantly associated with HRQOL in both men and women. Aging, underweight or obesity, and divorced or never married states were associated with lower HRQOL; higher education and household income levels were associated with higher HRQOL; high-risk drinking and physical activity were associated with higher HRQOL; smoking was associated with higher HRQOL in men, but lower HRQOL in women; and chronic diseases including hypertension, diabetes, and arthritis were associated with lower HRQOL.

In models I, II, and III, as well as in the full model, individual-level perceived stress and depression were significantly related to HRQOL in both men and women. Higher perceived stress and depression were associated with lower HRQOL. Regional stress in model II and regional depression in model III were also significant factors in both men and women. In the full model, regional stress was also a significant factor, whereas regional depression was not. Higher regional stress levels were associated with lower HRQOL in both men and women. The decrease in HRQOL from the first to the fourth quartiles of regional stress was greater in women (-1.09, 95% confidence interval [CI], -1.87 to -0.31) than in men (-0.65; 95% CI, -1.04 to -0.26). When compared to model II, the absolute value of regression coefficient of regional stress (first to fourth quartiles) on HRQOL attenuated by 14.0% and 5.4% in men and women, respectively, in the full model (Table 2).

## DISCUSSION

This study examined the associations of individual and regional-level perceived stress and depression with individual HRQOL measured in terms of the EQ-5D using a multilevel model. The ICCs calculated by the null model indicated regional variation in HRQOL among municipal districts, with greater variation among women than among men. Regional variation in HRQOL was mainly explained by individual-level variables according to the results of model I. Individual-level perceived stress and depression were significantly related to individual HRQOL. Regional stress and depression were also significant factors and both had greater negative impacts on HRQOL among women than among men.

The ICC of the null model (0.017 in men and 0.027 in women) in this study implies that the contribution of regional-level factors to HRQOL is relatively small. However, the small ICC does not mean that it is not worthwhile to investigate the effects of regional factors on individual-level HRQOL [9]. Although the contribution of regional-level factors is small, it may be a significant component of population-level approaches. In certain vulnerable groups, the impact may be greater, and further active exploration is therefore needed.

Compared to those with lower perceived stress and depression states at the individual-level, those with higher states had decreased HRQOL in this study, consistent with previous studies [23-27] that have reported negative effects of individual-level psychological states on health. The previous studies did not explore the role of regional-level psychological status. To the best of our knowledge, our study may be one of the leading reports showing the relationship between community-level stress and individual HRQOL through a multilevel analysis. Our results show that regional stress levels have an inverse association with HRQOL that is independent of individual-level perceived stress. Regional stress is thought to reflect environmental demands that are placed on local residents in common according to the heuristic model of Cohen et al. [20]. Our finding shows the need for research to attempt to identify specific environmental demands that affect regional psychological states.

In this study, the decrease in HRQOL from the first to the fourth quartiles of regional stress was greater in women than in men, perhaps due to gender differences in vulnerability, coping strategies, and social support [39-43]. Women have been reported to be psychologically and physiologically more vulnerable to stress exposure [41,44], and men and women are reported to approach psychological stress management in different ways [40]. One study suggested that women cope with stress in a less healthy way than men [43]. In our study, Korean women were found to have attained lower education levels than men, which can lead to maladaptive coping strategies and poorer access to social services. The higher levels of divorced or widowed status and lower income levels in Korean women in this study might also result in poorer access to social services compared to men.

The application of a multilevel analysis model in this study allowed us to simultaneously examine the effects of individual-level and regional-level indicators of psychological factors. Previous studies have often evaluated health levels using a single self-rated question [2,17,24,26,45]. An advantage of the present study design is that it used the EQ-5D, which evaluates health status across multiple dimensions, and the PHQ-9, which has been verified to have good reliability and validity, to evaluate depression instead of a single question.

This study had some limitations. First, since this was a cross-sectional study, the relationship between psychological factors and HRQOL cannot be confirmed as causal; a further longitudinal study is required.

Second, because the perceived stress variable of the KCHS was evaluated using only 1 question, we have no direct information on its reliability. However, perceived stress has been found to have stable relationships with socio-demographic characteristics, health behaviors, and chronic disease in several studies that have used KCHS data [46,47], so it is thought that this variable can be applied here.

Finally, without using directly measured environmental demands, we used only regional-level percentages derived from individual perceived stress or depression levels. To understand the role of regional psychological factors on individual HRQOL more fully, contextual variables that reflect environmental demands should be added to future analytical models.

In conclusion, our results indicate that regional stress and depression, as well as individual stress and depression, are inversely associated with individual HRQOL in both genders. Future research should consider the territorial characteristics of municipalities to identify the specific environmental demands that account for regional stress levels.

## Figures and Tables

**Table 1.** General characteristics of the study subjects

Characteristics	Men (n=99,819)	Women (n=116,894)	Total (n=216,713)	p-value
Age, mean±SD (yr)	52.4±17.0	53.3±17.3	52.9±17.1	<0.001
Body mass index				<0.001
Underweight	2,627 (2.6)	7,848 (6.7)	10,475 (4.8)	
Normal	63,559 (63.7)	83,087 (71.1)	146,646 (67.7)	
Overweight	29,845 (29.9)	22,983 (19.7)	52,828 (24.4)	
Obese	3,788 (3.8)	2,976 (2.5)	6,764 (3.1)	
Marital status				<0.001
Married	72,627 (72.8)	75,812 (64.9)	148,439 (68.5)	
Divorced/widowed	8,063 (8.1)	26,019 (22.3)	34,082 (15.7)	
Never married	19,129 (19.2)	15,063 (12.9)	34,192 (15.8)	
Education				<0.001
Less than middle school	14,189 (14.2)	32,905 (28.1)	47,094 (21.7)	
Middle school	11,737 (11.8)	13,574 (11.6)	25,311 (11.7)	
High school	31,521 (31.6)	31,593 (27.0)	63,114 (29.1)	
College or more,	42,372 (42.4)	38,822 (33.2)	81,194 (37.5)	
Household income (million KRW)				
<1.00	15,813 (15.8)	24,503 (21.0)	40,316 (18.6)	<0.001
1.00-1.99	15,712 (15.7)	18,768 (16.1)	34,480 (15.9)	
2.00-2.99	18,723 (18.8)	19,385 (16.6)	38,108 (17.6)	
3.00-3.99	16,814 (16.8)	17,999 (15.4)	34,813 (16.1)	
≥4.00	32,757 (32.8)	36,239 (31.0)	68,996 (31.8)	
Residential area				<0.001
Urban	56,750 (56.9)	67,854 (58.0)	124,604 (57.5)	
Rural	43,069 (43.1)	49,040 (42.0)	92,109 (42.5)	
Current smoking				<0.001
Yes	35,409 (35.5)	3,462 (3.0)	38,871 (17.9)	
No	64,410 (64.5)	113,432 (97.0)	177,842 (82.1)	
High-risk drinking				<0.001
Yes	21,860 (21.9)	5,317 (4.5)	27,177 (12.5)	
No	77,959 (78.1)	111,577 (95.5)	189,536 (87.5)	
Physical activity				<0.001
Yes	27,015 (27.1)	22,304 (19.1)	49,319 (22.8)	
No	72,804 (72.9)	94,590 (80.9)	167,394 (77.2)	
Hypertension				<0.001
Yes	26,969 (27.0)	30,397 (26.0)	57,366 (26.5)	
No	72,850 (73.0)	86,497 (74.0)	159,347 (73.5)	
Diabetes				<0.001
Yes	11,920 (11.9)	11,483 (9.8)	23,403 (10.8)	
No	87,899 (88.1)	105,411(90.2)	193,310 (89.2)	
Arthritis				<0.001
Yes	7,361 (7.4)	24,915 (21.3)	32,276 (14.9)	
No	92,458 (92.6)	91,979 (78.7)	184,437 (85.1)	
Perceived stress				<0.001
Higher	22,887 (22.9)	28,704 (24.6)	51,591 (23.8)	
Lower	76,932 (77.1)	88,190 (75.4)	165,122 (76.2)	
Depression				<0.001
PHQ-9≥10	2,375 (2.4)	4,624 (4.0)	6,999 (3.2)	
PHQ-9<10	97,444 (97.6)	112,270 (96)	209,714 (96.8)	
HRQOL, mean±SD	95±11	92±13	94±12	<0.001

Values are presented as number (%). SD, standard deviation; KRW, Korean won; PHQ, Patient Health Questionnaire; HRQOL, health-related quality of life.

**Table 2.** Multilevel analysis weighted results of health-related quality of life among Korean men, and women

			Men				Women			
			Model I	Model II	Model III	Model IV	Model I	Model II	Model III	Model IV
Individual-level										
	Age		-0.113 (0.005)^***^	-0.113 (0.005)^***^	-0.113 (0.005)^***^	-0.113 (0.005)^***^	-0.180 (0.006)^***^	-0.180 (0.006) ^***^	-0.180 (0.006) ^***^	-0.180 (0.006) ^***^
	BMI (ref: normal)									
		Underweight	-4.246 (0.343)^***^	-4.243 (0.342)^***^	-4.244 (0.342)^***^	-4.242 (0.342)^***^	-1.613 (0.151)^***^	-1.615 (0.151) ^***^	-1.613 (0.151) ^***^	-1.616 (0.150) ^***^
		Overweight	0.044 (0.072)	0.047 (0.069)	0.045 (0.072)	0.047 (0.072)	-0.308 (0.084)^***^	-0.307 (0.084) ^***^	-0.309 (0.084) ^***^	-0.307 (0.084) ^***^
		Obese	-0.700 (0.146)^***^	-0.696 (0.146)^***^	-0.700 (0.146)^***^	-0.696 (0.146)^***^	-1.942 (0.252)^***^	-1.929 (0.252) ^***^	-1.936 (0.252) ^***^	-1.929 (0.252) ^***^
	Marital status (ref: married)									
		Divorced	-0.881 (0.152)^***^	-0.880 (0.116)^***^	-0.882 (0.151)^***^	-0.880 (0.151)^***^	-1.544 (0.118)^***^	-1.544 (0.117) ^***^	-1.546 (0.117) ^***^	-1.545 (0.114) ^***^
		Never married	-1.666 (0.105)^***^	-1.662 (0.105)^***^	-1.662 (0.105)^***^	-1.661 (0.105)^***^	-1.977 (0.115)^***^	-1.973 (0.114) ^***^	-1.969 (0.115) ^***^	-1.971 (0.114) ^***^
	Education (ref: less than middle school)									
		Middle school	2.306 (0.176)^***^	2.305 (0.176)^***^	2.305 (0.175) ^***^	2.305 (0.175)^***^	3.375 (0.151)^***^	3.376 (0.150) ^***^	3.372 (0.150) ^***^	3.376 (0.149) ^***^
		High school	2.849 (0.152)^***^	2.851 (0.152)^***^	2.847 (0.152)^***^	2.849 (0.152)^***^	3.258 (0.160)^***^	3.260 (0.158) ^***^	3.255 (0.158) ^***^	3.260 (0.157) ^***^
		College or more	2.921 (0.153)^***^	2.924 (0.153)^***^	2.920 (0.153)^***^	2.923 (0.153)^***^	2.580 (0.179)^***^	2.575 (0.177)^***^	2.573 (0.177)^***^	2.578 (0.177)^***^
	Household income (million KRW) (ref: less than 1.00)									
		1.00-1.99	3.533 (0.175)^***^	3.534 (0.173)^***^	3.530 (0.174)^***^	3.532 (0.174)^***^	3.098 (0.149)^***^	3.105 (0.148)^***^	3.101 (0.148)^***^	3.106 (0.148)^***^
		2.00-2.99	4.316 (0.167)^***^	4.321 (0.166)^***^	4.313 (0.167)^***^	4.318 (0.167)^***^	3.142 (0.153)^***^	3.150 (0.153)^***^	3.141 (0.152)^***^	3.151 (0.153)^***^
		3.00-3.99	4.500 (0.169)^***^	4.507 (0.168)^***^	4.497 (0.169)^***^	4.505 (0.169)^***^	3.113 (0.151)^***^	3.127 (0.150)^***^	3.115 (0.149)^***^	3.128 (0.150)^***^
		≥4.00	4.569 (0.164)^***^	4.582 (0.162)^***^	4.568 (0.164)^***^	4.580 (0.164)^***^	3.205 (0.151)^***^	3.220 (0.150)^***^	3.207 (0.149)^***^	3.223 (0.150)^***^
	Residential area: rural (ref: urban)		0.262 (0.098)^**^	0.184 (0.099)	0.228 (0.101)^*^	0.178 (0.100)	0.205 (0.125)	0.139 (0.122)	0.229 (0.109)^*^	0.117 (0.131)
	Current smoking (ref: no)		0.369 (0.064)^***^	0.369 (0.064)^***^	0.369 (0.064)^***^	0.370 (0.064)^***^	-0.492 (0.201)^*^	-0.495 (0.201)^*^	-0.496 (0.201)^*^	-0.493 (0.201)^*^
	High-risk drinking (ref: no)		0.791 (0.061) ^***^	0.790 (0.060)^***^	0.791 (0.060)^***^	0.790 (0.061)^***^	0.313 (0.111)^**^	0.318 (0.111)^**^	0.315 (0.111)^**^	0.318 (0.112)^**^
	Physical activity (ref: no)		1.250 (0.067)^***^	1.247 (0.067)^***^	1.248 (0.067)^***^	1.247 (0.067)^***^	1.325 (0.094)^***^	1.324 (0.094)^***^	1.326 (0.093)^***^	1.324 (0.095)^***^
	Hypertension (ref: no)		-0.769 (0.088)^***^	-0.767 (0.088)^***^	-0.770 (0.088)^***^	-0.767 (0.088)^***^	-1.117 (0.100)^***^	-1.113 (0.099)^***^	-1.115 (0.100)^***^	-1.114 (0.099)^***^
	Diabetes (ref: no)		-1.332 (0.122)^***^	-1.332 (0.122)^***^	-1.332 (0.122)^***^	-1.331 (0.121)^***^	-1.499 (0.156)^***^	-1.501 (0.156)^***^	-1.500 (0.156)^***^	-1.500 (0.156)^***^
	Arthritis (ref: no)		-6.291 (0.190)^***^	-6.286 (0.189)^***^	-6.289 (0.189)^***^	-6.285 (0.189)^***^	-6.110 (0.120)^***^	-6.105 (0.120)^***^	-6.106 (0.120)^***^	-6.106 (0.120)^***^
	Higher perceived stress (ref: lower)		-3.121 (0.106)^***^	-3.106 (0.105)^***^	-3.119 (0.106)^***^	-3.107 (0.105)^***^	-3.975 (0.092)^***^	-3.962 (0.091)^***^	-3.971 (0.091)^***^	-3.962 (0.091)^***^
	Depression (ref: PHQ-9<10)		-17.666 (0.630)^***^	-17.661 (0.628)^***^	-17.648 (0.629)^***^	-17.652 (0.629)^***^	-14.095 (0.382)^***^	-14.073 (0.382)^***^	-14.065 (0.381)^***^	-14.069 (0.380)^***^
Contextual level										
	Regional stress (%, higher) (ref: 1st quartile)^1^									
		2nd quartile		-0.255 (0.160)		-0.195 (0.180)		-0.153 (0.225)		-0.161 (0.302)
		3rd quartile		-0.521 (0.152)^**^		-0.445 (0.172)^**^		-0.854 (0.270)^**^		-0.821 (0.368)^*^
		4th quartile		-0.755 (0.165)^***^		-0.649 (0.197)^***^		-1.150 (0.222)^***^		-1.088 (0.399)^**^
	Regional depression (%, PHQ-9≥10) (ref: 1st quartile)^2^									
		2nd quartile			-0.156 (0.151)	-0.086 (0.157)			-0.124 (0.138)	-0.068 (0.271)
		3rd quartile			-0.382 (0.143)^**^	-0.158 (0.162)			-0.723 (0.146)^***^	-0.054 (0.344)
		4th quartile			-0.498 (0.183)^**^	-0.226 (0.196)			-0.750 (0.177)^***^	-0.191 (0.363)
ICC^3^			0.007	0.006	0.006	0.006	0.012	0.010	0.013	0.010

Values are presented as beta (standard error). BMI, body mass index; KRW, Korean won; PHQ, Patient Health Questionnaire; ICC, intraclass correlation coefficient.

1 Regional-level age-adjusted rates of perceived stress were calculated for 254 municipal districts and categorized into quartiles.

2 Regional-level age-adjusted rates of depression (PHQ-9≥10) were calculated for 254 municipal districts and categorized into quartiles.

3 In the null model, ICC was 0.017 for men and 0.027 for women.

* p<0.05,

** p<0.01,

*** p<0.001.
